# The Livingstone College

**Published:** 1897-01-09

**Authors:** 


### THE LIVINGSTONE COLLEGE.

In regard to a paragraph which recently appeared in THE HOSPITAL in regard to Livingstone College, Dr. Harford-Battersby writes to say that the college is not connected with the Church Missionary Society, and that no theological teaching is given there.

